# Building integrated pathways to independence for diverse biomedical researchers: *Project Pathways*, the BUILD program at Xavier University of Louisiana

**DOI:** 10.1186/s12919-017-0081-x

**Published:** 2017-12-04

**Authors:** Maryam Foroozesh, Marguerite Giguette, Kathleen Morgan, Kelly Johanson, Gene D’Amour, Tiera Coston, Clair Wilkins-Green

**Affiliations:** 1 Department of Chemistry, Xavier University of Louisiana, 1 Drexel Drive, New Orleans, LA 70125 USA; 2 Office of Academic Affairs, Xavier University of Louisiana, 1 Drexel Drive, New Orleans, LA 70125 USA; 3 BUILD Program Office, Xavier University of Louisiana, 1 Drexel Drive, New Orleans, LA 70125 USA; 4 Center for the Advancement of Teaching and Faculty Development, 1 Drexel Drive, New Orleans, LA 70125 USA

## Abstract

**Background and purpose:**

Xavier University of Louisiana is a historically Black and Catholic university that is nationally recognized for its science, technology, engineering and mathematics (STEM) curricula. Approximately 73% of Xavier’s students are African American, and about 77% major in the biomedical sciences. Xavier is a national leader in the number of STEM majors who go on to receive M.D. degrees and Ph.D. degrees in science and engineering. Despite Xavier’s advances in this area, African Americans still earn about 7.5% of the Bachelor’s degrees, less than 8% of the Master’s degrees, and less than 5% of the doctoral degrees conferred in STEM disciplines in the United States. Additionally, although many well-prepared, highly-motivated students are attracted by Xavier’s reputation in the sciences, many of these students, though bright and capable, come from underperforming public school systems and receive substandard preparation in STEM disciplines. The purpose of this article is to describe how Xavier works to overcome unequal education backgrounds and socioeconomic challenges to develop student talent through expanding biomedical training opportunities and build on an established reputation in science education.

**Program and key highlights:**

The National Institutes of Health (NIH)/National Institute of General Medical Sciences (NIGMS)-funded BUILD (Building Infrastructure Leading to Diversity) Program at Xavier University of Louisiana, *Project Pathways*, is a highly-innovative program designed to broaden the career interests of students early on, and to engage them in activities that entice them to continue their education towards biomedical research careers. Project strategies involve a transformation of Xavier’s academic and non-academic programs through the redesign, supplementation and integration of academic advising, tutoring, career services, personal counseling, undergraduate research training, faculty research mentoring, and development of new biomedical and research skills courses. The Program also focuses on mentor training and providing faculty members with opportunities to improve their teaching skills as well as their research competitiveness. In addition to the wide range of activities supported by BUILD within the institution, Xavier University is partnering with a number of major research universities across the nation to achieve *Project Pathways’* goals.

**Implications:**

The strategies developed by *Project Pathways* are designed to address the challenges and barriers Xavier students face as they work towards graduate studies and entering the biomedical workforce. Xavier University of Louisiana has a long history of providing high quality, rigorous education to African American students in a very supportive environment with highly dedicated faculty and staff. The program highlighted here could be used by other institutions as a model program for assisting students in STEM and other biomedical fields of study to successfully matriculate through college and graduate school and develop their research careers.

## Institutional context and building on previous efforts

### Description of campus

Xavier University of Louisiana (Xavier) has a mission and history of inclusion in the education of underserved populations. Grounded in the Christian principles exemplified by its founders, Saint Katharine Drexel and the Sisters of the Blessed Sacrament, Xavier is the only Catholic and Historically Black College or University (HBCU) in the U.S. and has the distinction of being the only Catholic university founded by an American-born saint. [1] True to its HBCU roots, Xavier’s mission continues to be to create a more just and humane society by preparing its students to assume roles of leadership and service in a global society. Xavier is dedicated to a curriculum that focuses on the liberal arts, ethical and moral values, critical thinking, and career preparation for further graduate or professional school education.

The fall 2016 enrollment at Xavier University of Louisiana was 2997, 2328 of whom were undergraduates. Approximately 56.4% of Xavier’s students are from Louisiana, primarily from the New Orleans area. The remaining 43.6% come from other states, the District of Columbia, the Virgin Islands and abroad. Xavier’s student body is predominantly African American (72.7%), but the University is open to all.

Nationally recognized for its science, technology, engineering and mathematics (STEM) curricula, a Xavier education features a solid liberal arts foundation and broad learning experiences that extend beyond the classroom and campus [2, 3]. The University is best known for its reputation in the health professions, ranking first in the number of African Americans who earn Bachelor’s degrees in the physical sciences and second in the number earning Bachelor’s degrees in the biological and biomedical sciences [2]. A report released by the National Science Foundation in 2013 confirms Xavier’s success in educating science graduates. According to the report, Xavier is ranked first in producing African American graduates who go on to receive life sciences Ph.D. degrees; fifth in the Nation in producing African American graduates who go on to receive science and engineering Ph.D. degrees; and seventh in producing African American graduates who go on to receive physical sciences Ph.D. degrees [4].

Additionally, Xavier is currently third among the Nation’s colleges and universities in the number of African American graduates enrolled in medical school, according to data compiled by the Association of American Medical Colleges [5]. Xavier alumni contribute significantly to the number of underrepresented minority physicians, pharmacists, dentists, and other professionals in medically-related fields who practice throughout the United States, many of whom opt to work in the greater New Orleans area. The success of Xavier’s pre-medical program was also highlighted in *The New York Times* [3].

### History of past/current student research training programs

Xavier recognizes the value of hands-on research training and direct engagement of students in research projects and has a long history of providing research training opportunities to its students through programs funded by various federal agencies or private foundations. The University’s success in securing funding for these programs, and the institution-wide participation in them, demonstrate faculty and staff grant writing and management skills, their knowledge in designing and implementing innovative interventions, and their deep commitment to improving student training and educational opportunities. Students also articulate faculty commitment and structured research opportunities as critical to their development as scientists (see Xavier student examples, [6]).

## *Project Pathways*: The Xavier BUILD program

### Preparing students for biomedical careers

#### Evidence base for activities

Minorities currently represent an expanding portion of the U.S. population, and yet many groups are underrepresented in the STEM workforce. As reported in the 2016 U.S. News/Raytheon STEM Index, the number of White students who earned STEM degrees grew 15% in the last 5 years, while the number of Black students fell by about the same percentage. [7] In general, the Index showed that women, African Americans and Hispanics still lag far behind White and Asian men in earning degrees and securing jobs in the STEM fields. Unless scientific education becomes more inclusive, the U.S. will be denied the talents of a large segment of its population. A diverse biomedical workforce provides benefits to society by tapping into unique perspectives that, among other benefits, may help narrow the health gap with a focus on health inequities and disparities, while promoting and ensuring fairness.

Xavier is already a leader in placing African American students in professional and graduate programs in spite of the fact that it is a relatively small school, and the majority of its students and their families are socioeconomically challenged. Xavier does not require its students to report their financial status to the University, however, institutional data from the past 5 years indicates that an average of 43% of its students are in receipt of Pell grants, and about 32% are first generation college students. [8] Notably, Xavier was recently highlighted in a New York Times article for its sixth place ranking among all colleges and universities nationwide for the upward economic mobility of its students from the bottom fifth of the income distribution to the top three-fifths. [9] Even with this positive economic trend for Xavier’s students, there is always room for improvement.

Consistently, the HERI CIRP Freshman Survey indicates that approximately 50% of incoming freshmen enter Xavier with a ‘strong sense of belonging to a community of scientists’ and approximately 65% of incoming students indicate that they will definitely or probably pursue a STEM-focused career. Further, less than 5% of Xavier’s incoming freshmen say that the highest degree they plan to attain is a Bachelor’s degree; of the remainder, 10% intend to go on to a Master’s, 34% to a medical degree, 26% to a Ph.D. and a further 19% to a professional doctorate. Sixty-seven percent of Xavier’s current undergraduates are majoring in a STEM discipline. Therefore, Xavier, with its unique culture and brand in STEM fields and the largest proportion of its students with an interest in STEM, has the potential to lead the way to making a difference.

The national literature identifies unique barriers faced by African Americans and other individuals from underrepresented groups entering into biomedical research careers, including the lack of: (1) *Early Awareness and Deepening Exposure* to the type of rewards associated with biomedical research careers, and the sense of accomplishment gained from success in early STEM educational experiences; (2) *Supportive Relationships*, particularly those related to faculty advising and mentoring; (3) *Suitable Educational Infrastructure*, namely, (a) innovative STEM courses that engross students in activities that promote a STEM mindset, and (b) the supplemental help needed when they face educational challenges; and (4) *Active Engagement* in meaningful biomedical research experiences and the presence of faculty and institutional resources needed to do this. [10–13]

Recent surveys of Xavier life sciences alumni and the graduate faculty at other institutions who teach them (made possible by a NIH-funded BUILD planning grant) echoed many of these findings and pointed to areas in Xavier’s curricula and support services that needed to be revised or supplemented. The *Project Pathways* Program was designed to implement strategies focused especially on the 78% of Xavier’s pre-pharmacy/pre-medical students who change their career goals before the junior year, at which point obtaining the education and experience needed to pursue a biomedical Ph.D. becomes more difficult. This program aims to broaden the career interests of these and other students early on, and engage them in activities that encourage, support, and prepare them to continue their education towards biomedical research careers.

#### Program aims and activities

*Project Pathways* is a collaborative program supported by the combined, coordinated efforts of the Institutional Development Core (IDC), Student Training Core (STC), and Research Enrichment Core (REC). The Administrative Core oversees the activities of all Cores, and also provides support to enhance faculty research competitiveness. The Student Training, Research Enrichment, and Institutional Development Cores, together, have activities designed for students from freshmen to seniors, in addition to select recent Xavier graduates. These activities were developed to address the challenges and barriers Xavier students face in their path to the biomedical workforce, effectively increasing their graduation rates and improving their success rates in research careers.

#### Implementation plan

*Project Pathways* is a multi-pronged program with many activities that aim to enhance the academic and research infrastructure at Xavier for all students, and especially those who may go on to biomedical research careers. The Program provides resources for key offices and centers across the campus that assist students with academic support, professional development, and undergraduate research activities. *Project Pathways* has contributed to the expansion of both personnel and support of *the Student Academic Success Office* (SASO), *the Office of Career Services* (OCS), the *Center for Undergraduate Research and Graduate Opportunity* (CURGO), and the *Center for the Advancement of Teaching and Faculty Development* (CAT + FD).

A major goal of the Program is to increase the pool of current Xavier students who are interested in pursuing biomedical research careers, and expanding the number of biomedical research opportunities offered to Xavier students while also providing expanded academic support. The Program coordinates a number of activities designed to educate all interested freshman and sophomore students about the variety of possible biomedical research careers available, and helps them identify careers that match their interests. Program activities allow students to gradually and voluntarily increase their involvement, based on their level of interest in biomedical research. Both freshmen and sophomores are given opportunities to explore research labs at Xavier. The “Freshman Open House” allows freshmen to visit different research labs and is held during the *Biomedical Week* at Xavier, while sophomores can apply to shadow a current research student on campus. The shadowing experience allows students to see what the day-to-day activities are like in a research environment, as well as talk with research students and faculty about their projects and goals. The Program also facilitates a series of peer-led discussion groups where interested students, primarily sophomores, discuss science in the news (e.g., Ebola, Zika) and the various careers of people involved in the studies. They also hear from other students about their specific research projects and their experiences with the graduate school application process. This peer approach is critical to building and sustaining interest in science careers. [14] During the Biomedical Week, a number of seminars and activities are organized for Xavier students to increase their knowledge and interest in biomedical disciplines, such as a tour of the New Orleans Bioinnovation Center, and an informational session with representatives from a major pharmaceutical company.

Each spring, the Program chooses 12 sophomore students from among the participants in these different activities to become BUILD Scholars. The Scholars receive 2 years of mentored research, including a summer at a research-intensive partner institution, a monthly stipend, up to 60% of Xavier tuition, and funds for travel and research supplies. Mentors may also receive some academic year salary support and/or summer salary. In order to increase the reach of the Program beyond what was already included in the funded proposal, each year, several additional students are chosen as BUILD Research Students. Like the Scholars, the BUILD Research Students are matched with mentors and receive a monthly stipend, and funds for travel; however, due to budget limitations, a BUILD Research Student’s appointment is for a period of 12 months with potential for competitive continuation for an additional year. Additionally, these students do not receive tuition assistance or supply funds. The BUILD Research Students either applied to be a BUILD Scholar and were competitive, but not chosen, or were selected by a faculty member who was awarded funding for a pilot project (*vide infra*).

The Program also focuses on strengthening the supportive environment needed for Xavier students to overcome barriers to success through curricular enhancement, faculty mentor and student mentee training workshops, and post-baccalaureate research training opportunities for recent graduates. Several new courses have been developed with support from the Program including a *Science and Technical Writing* course, a *Scientific Communication* course, and an *Ethical Conduct in Scientific Research* course. The Program provides curriculum development mini-grants for faculty to develop new courses or modify existing courses in order to fill gaps in the biomedical curricula at Xavier.

To introduce the new BUILD participants to research, the Program coordinates the Entering Research workshop series for BUILD Scholars and BUILD Research Students. The curriculum, based on a successful program at the University of Wisconsin-Madison, involves helping students become aware of and acquire a number of soft skills necessary for becoming contributing members of a research group. [15] Topics covered include, but are not limited to: choosing a mentor, mentor-mentee communication, searching the literature, reading scientific articles, establishing goals and expectations, defining hypothesis or research question, and designing scientific experiments. The first three introductory modules are covered in the spring semester following student selection. The remaining workshops are held over the students’ first summer in the Program. The workshops are immediately followed by three workshops on writing small grant proposals offered through CURGO.

An aspect of *Project Pathways* showing early success, the BUILD Technician Program, is a post-baccalaureate research training program which provides recent Xavier graduates with additional training and research experience needed to successfully enter and complete graduate work. It is not uncommon for senior-level undergraduates to express a first-time interest in graduate school, and this program gives such students the time to become competitive applicants for admission to and completion of biomedical graduate programs. Others joining the Program as BUILD Technicians are students who have applied unsuccessfully to graduate school. BUILD Technicians participate in the Program for approximately 1 year, during which time they are considered full-time staff employees receiving all staff benefits. Additionally, they participate in BUILD enrichment activities such as GRE workshops and attend and present at scientific meetings. The success rate for the first cohort of BUILD Technicians entering graduate programs was 100%, and the Program is hopeful in continuing this trend.

### Developing faculty and support staff

To improve the quality of undergraduate research mentoring at Xavier, the Program provides training activities for faculty and staff, which educate Xavier research mentors on the challenges faced by their mentees and equip them to provide the necessary support both in and out of the laboratory. Xavier’s Center for the Advancement of Teaching and Faculty Development (CAT + FD) works closely with the National Research Mentoring Network (NRMN) (Sorkness et al., this volume) to present workshops on topics such as cultural awareness, stereotype threat, setting reasonable goals and expectations, and using technology to foster the mentor-mentee relationship.

To assure engagement of Xavier students in quality research experiences, it is also important to have a faculty who are research competitive. Consequently, it is necessary to address the need for junior faculty mentoring and release time, the need to expand Xavier’s collaborative research network, and to expose Xavier’s undergraduates to a broader range of research opportunities mentored and guided by faculty at Xavier and partner institutions. Pilot project funding is provided to address these needs. *Project Pathways* partners with two other faculty research programs at Xavier (Louisiana Cancer Research Consortium (LCRC) and NIH-funded Research Centers at Minority Institutions (RCMI)) to leverage the available funds and provide funding to a larger group of faculty members than would be possible without the partnership. Applicants/awardees fall into one of the following three categories: (1) recently hired faculty who need start-up funding; (2) more experienced investigators with current or recently terminated external grant support who are seeking bridge funds to keep their laboratories functioning while they seek longer term external funding; or (3) faculty who need seed funding to pursue new directions in their research. Faculty with program funds are expected to expand the network of research opportunities by working with BUILD Scholars, BUILD Research Students, BUILD Technicians, and/or other Xavier undergraduates seeking research experiences. Grant recipients are also expected to develop a well-defined student research experience. Where applicable, funded faculty are also expected to identify a collaborator from a partner institution who could also host Xavier students in their laboratory during the summer. In addition, the Xavier investigator is required to participate in BUILD-related activities such as mentor training workshops, grant writing workshops, and relevant inter-institutional research seminars.

### Institutional/infrastructure development

#### Program aims and activities

*Project Pathways* has extensively focused on improving and expanding Xavier’s instructional, advising, career development, research capacity, and faculty development infrastructure and environment. These resources are available to all members of the Xavier community, not only students and faculty involved in the *Project Pathways* Program. Xavier’s well-documented leadership in producing African American scientists has long been credited to both their academic preparation combined with the formative support these students receive from the academic and non-academic departments and offices on campus.

The professional staff from the Center for Undergraduate Research and Graduate Opportunity (CURGO) and Office of Career Services (OCS) help to prepare students to define and reach their career goals. This especially holds true for undergraduate students majoring in STEM disciplines who comprise 76% of Xavier’s student body. Xavier’s signature professional programs in pre-medicine and pre-pharmacy are very popular because students easily identify with the aforementioned careers and clearly see the pathway to and benefits of, success. However, many of Xavier’s students are unaware of biomedical careers outside of medicine and pharmacy. Through a series of broad-based workshops funded by the *Project Pathways* Program, CURGO and OCS assist Xavier students, beginning in the freshman year, in gaining a better understanding of the nature of biomedical research careers. Early exposure to biomedical research career paths allows students the opportunity to build the skills necessary for success. In addition, when motivated to pursue a specific career, students are more likely to engage in their courses, which leads to enhanced retention and improved graduation rates. [16] The major goal of these offices is to guide students through a process that results in their ability to identify a specific biomedical research interest, obtain experiences that advance their research skills, and develop the tools needed to be accepted to and succeed in graduate programs. They also offer assistance to students in creating Individual Development Plans, as well as assistance in preparing applications, resumes and *curriculum vitae*, and personal statements. To provide Xavier students with undergraduate research opportunities on and off campus, CURGO utilizes *GOLD RUSH EXPRESS*, Xavier’s online career services platform, as well as one-on-one support in identifying and applying to research opportunities. CURGO also sponsors student workshops on applying to summer programs, preparing for summer research experiences, and proposal writing. In addition to workshops and seminars, CURGO provides funding to students and faculty for research and travel to conferences. CURGO hosts the annual *Festival of Scholars* and *Summer Research Symposium*, which encourage Xavier students to present their research and creative work to the broader campus community.

The mission of Xavier’s Student Academic Success Office (SASO) is to improve retention and graduation rates of Xavier students by offering academic support through tutoring, supplemental instruction (SI), and academic success workshops. These services were originally offered only to students taking introductory courses in biology, chemistry, physics, mathematics, writing and reading. Using *Project Pathways* support, SASO has expanded its tutoring services and supplemental instruction to include sophomore- and junior-level courses. SASO also provides academic monitoring of all students using an Early Alert System, which prompts the faculty three times per semester to report any student academic, attendance, or behavioral issues. The alerts are then sent to the department heads and academic advisors who, in turn, meet with the students to assist them in overcoming the underlying issues, ideally, before their grades suffer.

Because effective mentoring is at the heart of Xavier’s *Project Pathways*, the Center for the Advancement of Teaching and Faculty Development (CAT + FD) provides specific support to faculty in developing and nurturing their mentoring relationships through the P-MAX (Preparing Mentors and Advisors at Xavier) Program. P-MAX is based on the Entering Mentoring seminar developed at the University of Wisconsin-Madison [17] and is designed to provide participating faculty and staff with the knowledge and skills needed to mentor and advise undergraduate students, particularly those engaged in research. The Program addresses topics such as mentor-mentee communication, goal- and expectation-setting, cultural competence, issue identification and resolution, and best practices for good mentoring and advising. Faculty are also provided a repository of resources to support their growth as mentors. P-MAX begins with an intensive, day-long, workshop followed by at least three additional one-hour workshops during each of the subsequent fall and spring semesters. Case studies (found in literature or based on faculty experiences) are routinely used to stimulate discussion on the topic to be addressed, and faculty are encouraged to bring their own real-world experiences for discussion. P-MAX programming is made accessible, relevant and inviting and is offered in varying formats (panel discussions, hands-on workshops, seminars), at different locations (program locations vary across campus and online) and by expert presenters/facilitators (outside speakers and consultants are frequently brought in to conduct workshops and seminars and advise CAT + FD staff). All research staff working directly with undergraduate research students are also expected to participate in the P-MAX workshops. Since the CAT + FD supports the development of Xavier’s faculty across all career stages and areas of professional responsibility, faculty are also supported in their teaching, scholarship, service, and work/life balance.

The Science Education Research Group (SERG) is an open forum for faculty to meet bi-weekly to bring questions, concerns or suggestions related to teaching and learning for discussion with each other. SERG meetings are informal and multidisciplinary to encourage and support collaboration and communication among and between science and non-science faculty. Topics discussed are suggested by the faculty participants, and discussions are facilitated by the *Project Pathways’* Education Improvement Specialist (EIS), who is housed in CAT + FD. The EIS also provides support in the form of topic research and presentation of pedagogical trends.

#### Curricular innovations

To strengthen students’ ancillary skills that foster success in research careers, *Project Pathways* has enabled the development or improvement of courses and workshops that increase their knowledge and ability to use research skills. Examples are: the *Entering Research* workshop series (a 10-session program), the *Library Research Techniques* workshop, workshops on *Writing Small Research Proposals*, the *Science and Technical Writing* course, the *Scientific Communication Skills* course, and the *Ethical Conduct in Scientific Research* course. The courses are open to all Xavier students. In addition to supporting efforts to increase the retention and success of Xavier students in the biomedical sciences and to assure courses taken by students are providing effective learning experiences, the Program provides funding and support for evaluation and revision/development of science and mathematics courses. Results from senior comprehensive exams are used by the various departments to identify areas of weakness in the biomedical curricula at Xavier. For many years, several of the introductory science courses have tested students’ vocabulary to improve students’ performance on standardized tests. *Project Pathways* has also provided support to update these vocabulary instructional materials, and in case of the material used in the Organic Chemistry course sequence, include words that help students prepare specifically for the GRE.

### Research/pipeline partnerships

As previously mentioned, pilot project funding is provided by the Program to increase faculty research competitiveness. It is important to note that the proposals submitted for this funding mechanism are peer-reviewed by outstanding external scientists and are approved by the NIH, and awards have rigorous reporting requirements. In addition, a number of partnerships have been forged to provide collaboration opportunities between Xavier faculty members and faculty at research-intensive institutions. These collaborations also help to ensure that Xavier students, including the BUILD Scholars, have opportunities for research training at such institutions. Currently, *Project Pathways* has formal partnerships with 18 research institutions. Each partner institution has a designated liaison to *Project Pathways* who participates in monthly conference calls with the BUILD Management Team. Through close collaboration with the liaisons, a number of Xavier students have been placed in summer research programs at partner institutions. Further, we are seeking to expand the number of Xavier students whose primary mentor is at one of our local partner institutions. New research collaborations between the faculty at Xavier and partner institutions have also been initiated. In the near future, faculty at partner institutions who mentor BUILD Scholars will be required to participate in an online version of Xavier’s P-MAX training or the equivalent at their university, if offered. In addition to benefiting the BUILD Scholars, this training will help faculty at majority institutions better understand how best to mentor not only students from underrepresented groups, but all students.

## Site-level evaluation design

Due to the very complex nature of the BUILD programs across the country, an extended and interlinked evaluation system has been put in place to thoroughly assess and evaluate each individual BUILD program while assessing the success and national contributions of the overall NIGMS BUILD initiative (see McCreath et al., this volume). Internal and external evaluation teams have collaborated with each other and the Coordination and Evaluation Center (CEC) at the University of California Los Angeles (UCLA) to ensure that program goals are accomplished and deadlines are met. External evaluation of *Project Pathways* is performed by the Office of Educational Innovation and Evaluation (OEIE) at Kansas State University, College of Education. Focusing on both formative and summative evaluation methods, Xavier’s evaluation team continues to develop predictive analytic plans as more data become available for analysis.

## Exposure in a unique, staged approach

Students’ path through the BUILD pipeline at Xavier focuses on tracking student success at critical stages of transition and marshals support for their success:

### Level 1

First, getting oriented to Xavier’s ethos and expectations in STEM at an early stage is critical for students and faculty. As students and their parents attend the *Freshman Orientation* meetings, the Office of Career Services invites them to a BUILD informational meeting at which basic program information and benefits, and the importance of biomedical research are presented to them. At the *Faculty Meet and Greet* session during the *Orientation Week*, research posters of current BUILD participants are also exhibited, highlighting undergraduate research at Xavier. All Xavier freshmen are required to take a two-semester *Freshman Seminar* course sequence, which the CURGO and OCS staff visit to provide the students with information about a variety of biomedical research careers and the resources provided by the BUILD Program, specifically. During the freshman and sophomore years, all Xavier students are invited to participate in informational meetings and roundtables on biomedical research careers. The students participating in these BUILD activities are considered Level 1 BUILD Students. In addition, all freshmen are encouraged to utilize the resources at the Student Academic Success Office.

### Level 2

Second, a peer approach is critical to sustaining engagement and maintaining student interest in research careers. [14] Students in the spring of their freshman year are invited to participate in peer-led discussion group meetings. During these meetings, the current BUILD participants present their research findings to the attendees or lead discussions on current STEM topics of interest. Both freshmen and sophomores are invited to visit active research labs on campus and/or shadow a current research student. Students are also invited to complete an Individual Development Plan through OCS. Sophomores taking advantage of these BUILD activities are considered Level 2 BUILD Students and can receive incentives for their participation.

### Level 3

Third, engagement with the Program is built with progressively more intense experiences. In fall of their sophomore year, students who have shown interest in biomedical research careers and attended the various Level 1 and Level 2 BUILD activities are eligible to apply to become BUILD Scholars (Level 3 BUILD Students). This is the first point in the Program where only selected students can participate. Each BUILD Scholar is matched with a BUILD Research Mentor, begins performing hands-on research, and attends the *Entering Research* workshop series the spring and summer after selection. BUILD Scholars stay in the Program until their graduation, receive tuition scholarships and monthly stipends in addition to travel and supply funds. BUILD Scholars are required to complete all BUILD training activities and courses. During the second summer as a BUILD Scholar, the students are required to participate in an off-campus research experience at a research-intensive university, preferably at one of the partner institutions. The summer research project for the student is expected to be in some way related to the academic year project, so that there is a sense of continuity. A number of competitive applicants not selected as BUILD Scholars are selected as BUILD Research Students. BUILD Research Students stay in the Program for 1 year with potential for renewal for a second year. They are matched with a research mentor, perform hands-on research, participate in the *Entering Research* workshop series, receive monthly stipends and funds for travel, and are required to participate in some BUILD training activities and courses.

### Post-baccalaureate

Finally, the Program also offers full-time research technician positions to recent graduates who have the potential and intent to continue their education in biomedically-relevant graduate programs, but who need additional training to become competitive. The BUILD Technicians receive funding and hands-on experience as research staff for a period of about 1 year while preparing for and applying to graduate school.

The Program offers opportunities to interested students to better prepare for graduate school and post-baccalaureate careers. Levels 1, 2, 3 and recently graduated students are encouraged to participate in GRE workshops provided by the Program, and take advantage of enhanced available career services.

## Potential contributions

Looking at the Program holistically, it is evident that its various initiatives touch every aspect of a student’s journey at Xavier from freshman year to graduation and beyond. The strong focus on evaluation makes this project a data-driven experiment expected to lead to the determination of effective practices which could be shared with the greater academic community with the ultimate goal of effectively increasing diversity in the biomedical workforce.
